# Near‐Complete Suppression of NIR‐II Luminescence Quenching in Halide Double Perovskites for Surface Functionalization Through Facet Engineering

**DOI:** 10.1002/advs.202403198

**Published:** 2024-06-26

**Authors:** Qiudong Duan, Yusheng Xu, Yu Zha, Fanju Meng, Qi Wang, Yugeng Wen, Jianbei Qiu

**Affiliations:** ^1^ Faculty of Material Science and Engineering Key Lab of Advanced Materials of Yunnan Province Kunming University of Science and Technology Kunming 650093 China

**Keywords:** double perovskites, emission mechanism, facet engineering, NIR‐II luminescence quenching, surface functionalization

## Abstract

Lanthanide‐based NIR‐II‐emitting materials (1000–1700 nm) show promise for optoelectronic devices, phototherapy, and bioimaging. However, one major bottleneck to prevent their widespread use lies in low quantum efficiencies, which are significantly constrained by various quenching effects. Here, a highly oriented (222) facet is achieved via facet engineering for Cs_2_NaErCl_6_ double perovskites, enabling near‐complete suppression of NIR‐II luminescence quenching. The optimally (222)‐oriented Cs_2_Ag_0.10_Na_0.90_ErCl_6_ microcrystals emit Er^3+^ 1540 nm light with unprecedented high quantum efficiencies of 90 ± 6% under 379 nm UV excitation (ultralarge Stokes shift >1000 nm), and a record near‐unity quantum yield of 98.6% is also obtained for (222)‐based Cs_2_NaYb_0.40_Er_0.60_Cl_6_ microcrystallites under 980 nm excitation. With combined experimental and theoretical studies, the underlying mechanism of facet‐dependent Er^3+^ 1540 nm emissions is revealed, which can contribute to surface asymmetry‐induced breakdown of parity‐forbidden transition and suppression of undesired non‐radiative processes. Further, the role of surface quenching is reexamined by molecular dynamics based on two facets, highlighting the drastic two‐phonon coupling effect of a hydroxyl group to ^4^I_13/2_ level of Er^3+^. Surface‐functionalized facets will provide new insights for tunable luminescence in double perovskites, and open up a new avenue for developing highly efficient NIR‐II emitters toward broad applications.

## Graphical abstract

The preferred (222) facet in Cs_2_NaErCl_6_ double perovskite is favorable for Er^3+^ 1540 nm emission with a record quantum yield of 98.6% under 980 nm excitation, which is useful for surface modification. These findings suggest facet engineering is a new way for realizing highly efficient Er^3+^‐based NIR‐II emitters toward broad applications.

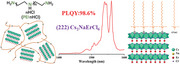

## 1 Introduction

Luminescent materials exhibiting emission between 700 and 1700 nm within the NIR region have been emerging in recent years since they are in demand for a range of present and next‐generation optical and optoelectronic applications including solid‐state lighting, solar cell conversion, NIR phosphor‐converted light‐emitting diode (pc‐LED), optical communication, as well as in vivo and in vitro imaging and sensing.^[^ ^1^, ^2^, ^3^ ^]^ Among them, lead‐free halide perovskites are considered promising luminescent candidates for greatly overcoming the instability and toxicity of lead halide perovskites.^[^ ^4^ ^]^ Specially, halide double perovskites (DPs) with the general formula A_2_M^+^M^3+^X_6_ possess a number of unique advantages, such as high stability, low toxicity, high wavelength tunability, and excellent photophysical properties, making them suitable for visible and NIR emissions.^[^ ^5^, ^6^, ^7^ ^]^ What is more, the octahedral coordination environment in DPs provides abundant opportunities for doping with lanthanide (Ln^3+^), transition metal ions (Mn^2+^ and Cr^3+^), and ns^2^ electrons of Bi^3+^ and Sb^3+^.

In particular, trivalent Ln^3+^ ions with unique 4f electronic configuration and rich energy levels can produce NIR light, covering from NIR‐I (700–1000 nm) to NIR‐II (1000–1700 nm) and beyond. For example, the photoluminescence quantum yield (PLQY) as high as 82.5% at 997 nm was reported for the Yb^3+^‐doped Cs_2_AgBiBr_6_ film.^[^ ^6a^ ^]^ Specially, an NIR‐II light at 1540 nm is needed because of its great promise in NIR photography, deep‐tissue, optical communication, and high‐resolution imaging.^[^ ^8^ ^]^ Although 1540 nm emission can be supplied by the ^4^I_13/2_→^4^I_15/2_ transition of Er^3+^, Er^3+^‐doped DPs inevitably suffer from poor PLQY due to low absorption coefficient, parity‐forbidden optical transition, and inefficient energy transfer. It has thus remained a challenge to obtain bright Er^3+^ 1540 nm emission. Doping ions can be an effective method for improving NIR efficiency, yet the concentration of lanthanide ions should be carefully controlled because of concentration quenching. In particular, cross‐relaxation between lanthanide ions at high doping levels is historically regarded as the major course of concentration quenching,^[^ ^9^ ^]^ leading to luminescence quenching in Ln^3+^‐doped DP single crystals.

Crystal orientation correlates closely to the properties of materials for various applications such as solar cells, battery systems, and photocatalysis.^[^ ^10^, ^11^, ^12^ ^]^ However, it is quite difficult to tailor the facet orientation in DPs due to the uncontrolled crystal growth process. Currently, nearly all the DPs have the same (220)‐dominated facet,^[^ ^13^ ^]^ and thus it is impossible to investigate the impact of crystal orientation on their optical properties. However, from the viewpoint of facet orientation, it may be a new way to manipulate visible and NIR luminescence and provides an important insight into the photophysical pathways in DPs.

Here, we report that the preferred high‐index (222) facet is thoroughly favorable for Er^3+^ 1540 nm emission in Cs_2_NaErCl_6_ DPs under multiwavelength‐excitation, exhibiting a record quantum yield up to 98.6% under 980 nm excitation. In particular, we demonstrate the major deactivation pathway at high Er^3+^ concentrations is surface quenching via high‐energy vibrations of the surface OH^−^ group, providing a fundamental mechanistic understanding of various quenching pathways, which are commonly misunderstood in Er‐based microcrystallites.

## 2 Results and Discussion

**Figure 1:**
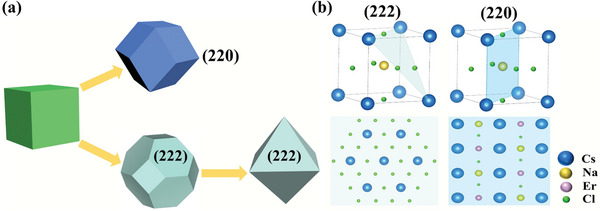
a) Schematic diagram of the evolution of (222) and (220) facets from cubic unit cell. b) Atomic arrangements of (220) and (222) facets on a perovskite cubic crystal.

The synthetic method of Cs_2_NaErCl_6_ DPs can be seen in the Supporting Information. **Figure** **1**a shows the evolution of two different facets from cubic unit cells. The growth rate is exponentially proportional to the surface energy of each crystal facet,^[^ ^14^ ^]^ and therefore the conventional DP crystals will be enclosed by (220) facets with minimum surface energy. Meanwhile, the crystallographic planes can also be controlled by the growth rate ratio along the (100) to that of (111),^[^ ^15^ ^]^ indicating the possible controllable growth of (111) facet. It should be noted that (111) and high‐index (222) facets are equally crystallographic orientations with similar truncated octahedral or octahedral shapes. It is expected that the surface properties of DP crystals would be different due to diverse atomic arrangement and coordination. Figure 1b exhibits atomic arrangements of (220) and (222) facets on a perovskite cubic crystal. The (222) facet can be terminated 3/8 Cs cation and 3/2 chloride anion, which are negatively charged. In contrast, (220) facet shows positively charged characters due to less chloride anions. Thus, this big difference is an essential prerequisite for tailoring the luminescent properties of DPs.

**Figure 2:**
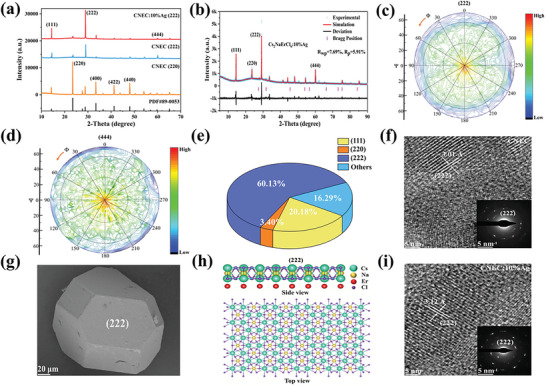
a) XRD patterns of undoped and 10% Ag^+^‐doped CNEC (222) and CNEC (220). b) XRD Rietveld refinement of CNEC:10% Ag (222). c,d) Normalized (222) and (444) pole figures of pristine CNEC (222). e) The facet distribution calculated from the average areal ratio for five CNEC:10% Ag (222) samples. f) HRTEM image and SAED pattern of pristine CNEC (222). g) SEM image of CNEC:10% Ag (222). h) Crystal structure of CNEC (222) in side view and top view. i) HRTEM image and SAED pattern of CNEC:10% Ag (222).

X‐ray diffraction (XRD) was used to evaluate the orientation of Cs_2_NaErCl_6_ DP. The Cs_2_NaErCl_6_ samples with favorable (220) plane, labeled as CNEC (220), show the same XRD patterns with the standard card (PDF#89‐0053) and as‐reported Cs_2_NaErCl_6_ DPs (**Figure** **2**a).^[^ ^16^ ^]^ Most impressively, (222) facet orientation is dramatically promoted after altering the growth rate, marked as CNEC (222), indicating that the preferred orientation turns to (222) from conventional (220). Similarly, Ag‐alloyed Cs_2_NaErCl_6_ DPs simplified as CNEC: Ag (222), don't change the oriented (222) crystal plane. The XRD Rietveld refinement results demonstrate that both pristine Cs_2_NaErCl_6_ and Ag‐alloyed Cs_2_NaErCl_6_ (222) samples still belong to the *Fm‐3m* space group and exhibit a highly symmetrical face‐centered cubic crystal structure (Figure 2b; Table S1, Supporting Information). The decreased lattice parameters after Ag doping are a result of stronger covalency between Ag^+^ and Cl^−^. XRD pole‐figure was further used to unveil the preferred facet distribution. The (222) peak intensity of pristine CNEC (222) is mainly located at the center, suggesting that (222) facet is one dominant plane (Figure 2c). Meanwhile, pole figures of (444) and (220) facets are also obtained (Figure 2d; Figure S2, Supporting Information) for roughly analyzing the crystal planes. As seen in Figure 2e, the percentage of (222) is calculated to be 60%, and the total percentage of (111) and (444) is estimated to be 30%. Thus, (222) facet and its equal orientations are dominate in controlled CNEC samples.

The modeling (222) crystal structure agrees well with those determined by electron microscopy (Figure 2h; Figure S3, Supporting Information). The scanning electron microscopy (SEM) images show that the products are micro‐sized crystals with uniform distribution of all the elements using energy‐dispersive X‐ray spectroscopy (EDS). The actual doping contents were determined from both EDS and the induced coupled plasma optical emission spectrometry in Table S2 and Figure S4 (Supporting Information). A truncated octahedral shape is observed based on CNEC:10% Ag (222), a typical shape of (111) or (222) facets in perovskites (Figure 2g). The high‐resolution transmission electron microscopy (HRTEM) images confirm a high crystallinity and the lattice spacing values are 3–3.1 Å, corresponding to the (222) lattice planes (Figure 2f–i). Meanwhile, all the selected area electron diffraction patterns exhibit polycrystalline rings, which can be attributed to the polycrystal characteristics of as‐prepared samples (Figure S5, Supporting Information).

**Figure 3:**
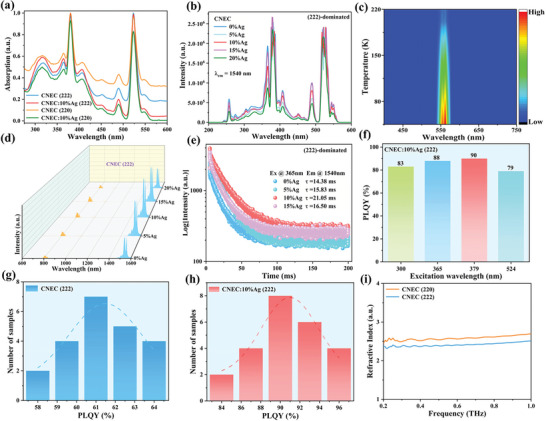
a) UV–vis absorbance spectra of undoped and 10% Ag^+^‐doped CNEC (222) and CNEC (220). b) PLE spectra of Ag‐alloyed CNEC (222) with different concentrations. c) Pseudo colormap of temperature‐dependent (80–220 K) PL in the visible range for CNEC:10% Ag (222) under 365 nm excitation. d) PL spectra of (222)‐dominated CNEC samples with different Ag^+^ contents under 365 nm excitation. e) The PL decay curves of Ag‐alloyed CNEC (222) with different doping concentrations under 365 nm excitation. f) The measured PLQY values for CNEC:10% Ag (222) under different excitation wavelengths. g,h) The statistical PLQY values for pristine CNEC (222) and CNEC:10% Ag (222) under 379 nm excitation, respectively. i) The refractive index measurements of pristine CNEC (222) and CNEC (220).

The optical properties of CNEC (222) and Ag‐alloyed CNEC (222) with different concentrations were systematically studied to check the facet effect. **Figure** **3**a shows UV–vis absorption spectra of undoped and 10% Ag^+^‐doped CNEC (222) and CNEC (220). All the absorption curves are similar with intense absorption peaks at 379 and 524 nm, arising from characteristic f–f transitions of Er^3+^. PL excitation (PLE) spectra are consistent with absorption spectra at 1540 nm for Ag‐alloyed CNEC (222) with different concentrations (Figure 3b). Although self‐trapped excitons (STE) typically exist in Ag‐based materials due to Jahn–Teller distortion, low‐temperature PL data present that narrow 554 nm emission corresponding to ^4^S_3/2_→^4^I_15/2_ transition of Er^3+^ is observed instead of broad STE emission for CNEC:10% Ag (222), indicating very weak electron‐phonon coupling (Figure 3c). Meanwhile, both green and red emissions are largely suppressed with increasing temperature based on (222) facets compared with (220) counterparts (Figure S6, Supporting Information), which can also be considered as another kind of “non‐radiative” emissions compared with desired 1540 luminescence. The short‐wave infrared emission shows main non‐asymmetrical peaks at 1540, 1547, and 1561 nm under 365 or 379 nm excitation, corresponding to Stark splitting ^4^I_13/2_ sublevels of Er^3+^ (Figure 3d; Figure S7, Supporting Information). Besides, only a weak 808 nm emission can be found in PL spectra, which can be assigned to the ^4^I_9/2_→^4^I_15/2_ transition of Er^3+^. The PL decay curves of Ag‐alloyed CNEC (222) with different concentrations were then measured (Figure 3e; Table S3, Supporting Information). The lifetimes of all the samples exhibit a single‐exponential function, with a maximum value of 21.05 ms for CNEC:10% Ag (222), in consistent with PLQY results (Figure S8, Supporting Information). Further increased Ag^+^ content would reduce lifetime, as an evidence of enhanced non‐radiative recombination rate.

Surprisingly, high PLQYs in the range of 1500–1700 nm can be obtained with different excitation wavelengths, including 300, 365, 379, and 524 nm (Figure 3f; Figures S9–S12, Supporting Information). In particular, the pristine CNEC (222) samples show the average PLQY of 61% (Figure 3g), in strikingly contrast to 24% on conventional (220) counterparts (Figure S13, Supporting Information), demonstrating strong facet effect on Er^3+^ 1540 nm emission in Cs_2_NaErCl_6_ DPs. The best‐performing CNEC:10% Ag (222) samples exhibit high quantum efficiencies of 90 ± 6% under 379 nm UV excitation (Figure 3h). The accurate refractive index is measured by QT‐TO1000 terahertz imaging system, as displayed in Figure S1 (Supporting Information). The refractive indexes are 2.54 and 2.38 for pristine CNEC (220) and CNEC (222), respectively. The refractive index is also different due to the anisotropic facets of Cs_2_NaErCl_6_ DPs.

**Figure 4:**
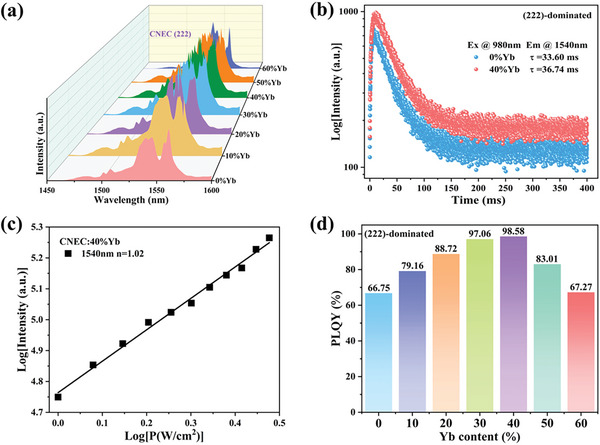
a) PL spectra of (222)‐dominated CNEC samples with different Yb^+^ contents under 980 nm excitation. b) The PL decay curves of CNEC (222) and CNEC:40% Yb (222) under 980 nm excitation. c) The power density dependence of 1540 nm emission for CNEC:40% Yb (222) under 980 nm excitation. d) The measured PLQY values for pristine CNEC (222) and Yb^3+^‐doped CNEC (222) under 980 nm excitation.

Meanwhile, obvious Er^3+^ 1540 nm luminescence can also be achieved for Yb^3+^‐doped CNEC (222) under 980 nm excitation. The corresponding characterizations can be seen in Figure S14 (Supporting Information). (222) facet enables high‐concentration doping of Yb^3+^, which is beneficial for bright emission (**Figure** **4**a). The fitting lifetimes of all the samples exhibit the single‐exponential function, with a maximum value of 36.74 ms for CNEC:40% Yb (222), as shown in the PL decay curves (Figure 4b; Figure S15 and Table S4, Supporting Information). PL decay curves at different emission wavelengths were also measured to better understand the excited‐state dynamics in Cs_2_NaErCl_6_ DPs (Figure S16 and Table S5, Supporting Information). The NIR‐II emission is originated from an energy transfer from Yb^3+^ to Er^3+^ because the emission intensity shows a linear relationship with the pump power of the 980 nm laser (Figure 4c). Furthermore, both pristine CNEC (222) and Yb^3+^‐doped CNEC (222) samples exhibit high quantum efficiencies under 980 nm excitation (Figure 4d; Figures S17 and S18, Supporting Information). Specially, CNEC:40% Yb (222) shows a near‐unity quantum yield of 98.6% under 980 nm excitation, which is the highest value for Er‐based halide perovskites, to the best of our knowledge (Table S6, Supporting Information). Moreover, the operational stability of DP emitters under continuous illumination with 379 and 980 nm has been measured as shown in Figure S19 (Supporting Information). The samples show good emission stability over 100 h with negligible degradation of PLQY, indicating their potential for broad applications.

The PLQY is defined as the ratio of the radiative recombination rate (*k_r_*) to the sum of the radiative and non‐radiative (*k_nr_*) recombination rates. Therefore, increasing *k_r_* and reducing *k_nr_* are two strategies to enhance the PLQY. As presented in **Table** **1**, *k_r_* of pristine CNEC (222) is much larger than that of CNEC (220), meaning the effective breakdown of parity‐forbidden transition by (222) facet. Considering the bulk centrosymmetric structure of all the Cs_2_NaErCl_6_ DPs, the origin of breaking symmetry must originate from surface asymmetry due to an imbalance charged surface. The built‐in electric field at (222) surface is beneficial for alleviating the ^4^I_13/2_→^4^I_15/2_ transition selection rule, promoting the structure asymmetry, and enhancing the luminescence efficiency. The lower symmetry has also been verified by the larger *Ω_2_* from Judd–Ofelt (J–O) analysis (Table 1).^[^ ^17^ ^]^ The higher value of *Ω_2_*/*Ω_4_* in CNEC:10% Ag (222) confirms stronger covalency after Ag^+^ doping, in accord with the Rietveld refinement results (Table S7, Supporting Information). In addition, CNEC:10% Ag (222) has a slightly larger *k_r_* compared with CNEC (222), indicating the same effect of (222) facet on the symmetry or the radiative recombination rate. The calculated transition probabilities by J–O analysis are in accordance with the *k_r_* values calculated by the fitted data of the time‐resolved PL decay, providing theoretical evidence that facet engineering can manipulate the symmetry for increasing the oscillator strength to improve the PLQY.

**Table 1:** Fitted average lifetimes, calculated *k_r_* and *k_nr_*, J–O parameters, and transition probabilities from J–O analysis.

Sample	*τ* [ms]	*k_r_* [ms^−1^]	*k_nr_* [ms^−1^]	*Ω_2_* [cm^2^]	Calculated probability [s^−1^]
CNEC (220)	14.94	1.61	5.09	2.35 × 10^−20^	674.92
CNEC (222)	14.38	4.24	2.71	3.76 × 10^−20^	1189.41
CNEC:10Ag%(222)	21.05	4.28	0.48	4.87 × 10^−20^	1199.68

On the other hand, the efficiency is usually limited by undesired competing non‐radiative processes, including multi‐phonon relaxation (MPR), surface quenching (SQ), cross‐relaxation (CR) and energy migration to defects (EM),^[^ ^18^, ^19^, ^20^, ^21^, ^22^, ^23^ ^]^ i.e., the total *k*_nr_ = *k_MPR_* +*k_SQ_* +*k_CR_* +*k_EM_*. Specifically concerning 1540 nm emission (≈6500 cm^−1^ of energy gap), *k_MPR_* is closely related to phonon‐mediated relaxation, and its contribution can be omitted due to the low phonon frequency of Cs_2_NaErCl_6_ DPs according to the “energy gap law” (Figure S20, Supporting Information).^[^ ^19^ ^]^ *k_EM_* is dependent on the density of defects. Regarding the lattice defects, they are very few in high‐crystallinity CNEC DPs as evidenced by ultra‐narrow diffraction peaks. Besides, antisite defects can also be quantitatively evaluated by the relative intensity ratio of (111) and (200) peak (I_111_/I_200_).^[^ ^5^ ^]^ As shown in Figure S21 (Supporting Information), CNEC:10% Ag (222) has the largest I_111_/I_200_, even better than that of the standard pattern of Cs_2_NaErCl_6_, suggesting a high degree of Na(I) and Er(III) site ordering and negligible lattice defects. Although cross‐relaxation is usually recognized as the main origin of concentration quenching, its effect (*k_CR_*) is less at high Er^3+^ concentrations in our case, only 1.5% for ^4^I_9/2_→^4^I_15/2_ transition under 379 nm excitation. Consequently, surface quenching (*k_SQ_*) should be responsible for the facet‐dependent quantum efficiency. As shown in Table 1, the calculated *k_nr_* of CNEC:10% Ag (222) is one order of magnitude slower than that of CNEC (220), proving that non‐radiative processes can be strongly suppressed by (222) facet.

**Figure 5:**
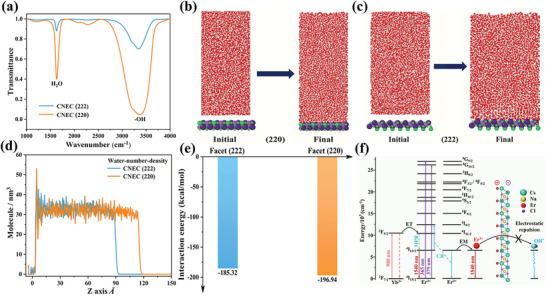
a) FTIR spectra of pristine CNEC (222) and CNEC (220). b,c) Molecular dynamics simulations of the interaction between H_2_O molecules and (220) and (222) facets, respectively. d) The distribution of water‐number‐density along the Z axis for (220) and (222) facets. e) The calculated interaction energy for (220) and (222) facets. f) Photophysical processes for Er^3+^ 1540 nm emission and proposed mechanism for bright NIR‐II luminescence in (222)‐dominated Cs_2_NaErCl_6_ DPs.

It is known that ^4^I_13/2_ emission of Er^3+^ is very sensitive to the presence of OH^−^ vibrations, due to energy resonance for strong quenching (energy gap of ≈6500 cm^–1 ^vs 3200–3700 cm^−1^ of O–H vibrations).^[^ ^24^ ^]^ However, compared to nanocrystals, surface quenching is commonly not considered the major quenching pathway in microcrystalline materials. In our case, we can infer that the predominant deactivation pathway is indeed a surface effect via the two‐phonon coupling of OH^−^ group. To validate this conjecture, Fourier transform infrared spectroscopy (FTIR) was used to investigate the surface information for two facets. **Figure** **5**a presents two characteristic vibrations at 1634 and 3430 cm^–1^, which can be attributed to bending vibrations of absorbed H_2_O molecules and stretching vibrations of O–H group. CNEC (222) exhibits much weaker vibration strengths of both H_2_O and OH^−^ than those of CNEC (220), indicating that (222) facet can effectively suppress the absorption of H_2_O and OH^−^. Thus, much lower PLQYs of CNEC (220) are expected via an intense two‐phonon relaxation process due to strong coordination with surface species.

Molecular dynamics simulations were further used to examine the interaction between H_2_O molecules and two facets (details in the Supporting Information). (220) and (222) facets were simulated as surfaces comparatively to interact with amounts of water molecules from the atomic level (Figure 5b,c). Figure 5d displays the simulation results of the distribution of water‐number densities along the Z axis. CNEC (220) surface has a higher density of water molecules, demonstrating its stronger interaction with H_2_O and OH^−^ species. The interaction energy per nm^2^ was also calculated based on the two systems. The resulting values are −196.94 and −185.32 kcal mol^−1^ for (220) and (222) facets, respectively (Figure 5e). From the calculation, it is clear that (220) is more energetically favorable for H_2_O molecules than (222) facet in terms of molecular dynamics. Both the experimental and theoretical evidences support that (222) facet can overcome the surface quenching from the coordinated water molecules.

The underlying mechanism is then proposed to explain the facet effects in Cs_2_NaErCl_6_ DPs. The energy level diagram is shown in Figure 5f. The excitation energy, for example, 379 or 980 nm, is well adsorbed by the ^4^G_11/2_ or ^2^F_5/2_ levels and then relaxed to ^4^I_13/2_ level for 1540 nm emissions. At high Er^3+^ concentrations, the CR process occurs inevitably, ^4^I_13/2 _+_ _^4^I_13/2_→^4^I_15/2 _+_ _^4^I_9/2_, although its contribution is low. Energy can diffuse or hop rapidly between Er^3+^ via energy migration, which may be released by the OH^−^ group from the surrounding environment. For conventional (220)‐dominated facet, the surface can absorb H_2_O molecules easily due to electrostatic interaction, leading to surface quenching for huge efficiency loss. In comparison, two significant benefits from (222) facet are electrostatic repulsion to OH^−^ and protection by the outside ion layer like a “core‐shell” structure. Therefore, the excitation energy can be preserved well and then converted efficiently to 1540 nm luminescence. Therefore, the studies elucidate the essence of surface quenching in Er^3+^‐based microcrystalline materials and offer an alternative strategy for near‐complete suppression of NIR luminescence quenching.

**Figure 6:**
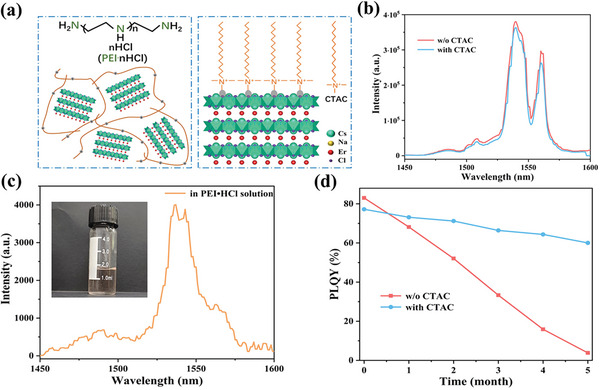
a) Schematic illustration of CTAC‐modified (222) facet and a mixture of CNEC and PEI·HCl. b) PL spectra of CNEC:50% Yb (222) samples with and without CTAC modification under 980 nm excitation. c) PL spectrum of CNEC:50% Yb (222) sample in PEI·HCl solution under 980 nm excitation. d) Stability tests of CNEC:50% Yb (222) samples with and without CTAC passivation in air for 5 months.

As proof, the negatively charged (222) surface shows surface functionalization via electrostatic interaction. It can be easily passivated by self‐assembled long‐chain organic amine for stable Er^3+^ 1540 luminescence or can be merged with amino polymer for NIR‐II emission in aqueous solution (**Figure** **6**a). The cetyltrimethylammonium chloride (CTAC)‐modified (222) facets show a little luminescence degradation (Figure 6b). Besides, CNEC:50% Yb (222) is mixed with the protonated form of polyethyleneimine (PEI·HCl) to produce weak Er^3+^ 1540 emission under 980 nm irradiation (Figure 6c). The transparent solution indicates that DP samples are protected well by the outside polymer in the acidic aqueous environment (inset of Figure 6c). What is more, the CTAC‐modified CNEC:50% Yb (222) samples exhibit good stability over 5 months, with degradation of PLQYs slowly (Figure 6d). In contrast, PLQYs of unmodified CNEC:50% Yb (222) samples drop to only 3% in the fifth month due to high hygroscopicity of Cs_2_NaErCl_6_, as evidenced by the contact angle tests (Figure S22, Supporting Information). The effective passivation of CTAC makes sure that (222)‐dominated CNEC can be further applied for broader in‐laboratory use and potential practical applications.

## 3 Conclusion

In summary, we have discovered anisotropic NIR‐II luminescence in lead‐free Cs_2_NaErCl_6_ double perovskites based on two different facets. The underlying mechanisms of facet‐dependent Er^3+^ 1540 nm luminescence are breaking the parity‐forbidden transition by the built‐in electric field at (222) surface for enhancing radiative recombination rates and suppressing various quenching processes for reducing non‐radiative recombination rates. The exact role of various quenching pathways in Er^3+^‐based perovskites can also been elucidated on (222) facet, including multi‐phonon relaxation, surface quenching, cross‐relaxation, and energy migration to defects. Moreover, surface quenching effects, mainly the two‐phonon coupling effect of hydroxyl group to ^4^I_13/2_ level of Er^3+^, show a direct impact on Er^3+^ 1540 nm emissions in microcrystalline materials, as evidenced by both experimental and theoretical studies. Our findings suggest facet engineering is a new way out of the existing technologies for realizing highly efficient Er^3+^‐based NIR‐II emitters, toward broad applications in high‐resolution fluorescence imaging, NIR LEDs, short‐wave infrared lasers, and beyond.

## Conflict of Interest

The authors declare no conflict of interest.

## Supporting information

Supporting Information

## Data Availability

The data that support the findings of this study are available in the supplementary material of this article.
