# Supplementary material for: Near‐Complete Suppression of NIR‐II Luminescence Quenching in Halide Double Perovskites for Surface Functionalization Through Facet Engineering
Source: Adv Sci (Weinh). 2024 Jun 26;11(32):2403198. doi: 10.1002/advs.202403198 (PMC11348257; doi:10.1002/advs.202403198)
Supplement: Supplementary file 1 — Supporting Information [file ADVS-11-2403198-s001.docx]

**Supporting Information**

**Near-Complete Suppression of NIR-Ⅱ Luminescence Quenching in Halide Double Perovskites for Surface Functionalization through Facet Engineering**

*Qiudong Duan, Yusheng Xu, Yu Zha, Fanju Meng, Qi Wang, Yugeng Wen,* and Jianbei Qiu**

______________________________________________

Q. Duan, Y. Xu, Y. Zha, F. Meng, Q. Wang, Y. Wen, J. Qiu

Faculty of Material Science and Engineering

Key Lab of Advanced Materials of Yunnan Province

Kunming University of Science and Technology

Kunming 650093, China

E-mail: wenyg@kust.edu.cn; qiu@kust.edu.cn

**Experimental Section**

***Materials***: Cesium chloride (CsCl, 99.99%), erbium oxide (Er_2_O_3_, 99.99%), silver chloride (AgCl, 99.99%), sodium chloride (NaCl, 99.99%), ytterbium chloride hexahydrate (YbCl_3_·6H_2_O, 99.99%), polyethyleneimine (PEI), cetyltrimethylammonium chloride (CTAC) and hydrochloric acid (HCl, 37wt% in water) were purchased from Shanghai Aladdin Biochemical Technology Co., Ltd. All materials and chemicals were used without further purification.

***Synthesis and surface modification***: The Cs_2_NaErCl_6_ microcrystals were synthesized by the hydrothermal method similar to the previous procedures with a change of cooling speed.^(1)^ Briefly, 4 mmol CsCl, 2 mmol NaCl, and 1 mmol Er_2_O_3_ were mixed in 15 mL HCl in a 50 mL Teflon liner. The mixture solution was heated at 180 ℃ for 12 h in a stainless-steel Parr autoclave, and was then cooled down slowly to room temperature at a speed of 10 ℃/h. The precipitated crystals were then filtered out, washed with anhydrous ethanol, and vacuum dried. The synthesis of Ag-alloyed Cs_2_NaErCl_6_ crystals were similar to Cs_2_NaErCl_6_ crystals. Typically, 4 mmol CsCl, 4 mmol NaCl, 2–x mmol Er_2_O_3_, and x mmol AgCl were mixed in 15 mL HCl in a 50 mL Teflon liner and then following the same procedure as for the Cs_2_NaErCl_6_ crystal synthesis.

The (222)-oriented Cs_2_NaErCl_6_ microcrystals were synthesized by a hydrothermal method. Briefly, 4 mmol CsCl, 2 mmol NaCl, and 1 mmol Er_2_O_3_ were mixed in 15 mL HCl in a 50 mL Teflon liner. The mixture solution was heated at 180 ℃ for 12 h in a stainless-steel Parr autoclave and was then cooled down to room temperature naturally. The precipitated crystals were then filtered out, washed with anhydrous ethanol, and vacuum dried. The synthesis of Ag-alloyed (222)-oriented Cs_2_NaErCl_6_ microcrystals were similar to Cs_2_NaErCl_6_, in addition to AgCl with different concentrations. The synthesis of Cs_2_NaEr_1-x_Yb_x_Cl_6_ microcrystals were similar to Cs_2_NaErCl_6_ with different concentrations of YbCl_3_·6H_2_O.

CTAC was firstly dissolved in anhydrous methanol, and was then mixed with DP samples for treatment. The mixture of protonated PEI and concentrated HCl was stirred at 60 ℃ for 6 h to get a well-mixed product, and then cooled down to room temperature naturally. The resulting products can then mix with DP samples for further use.

***Characterization***: The powder XRD patterns were identified using a laboratory powder XRD system at a scanning rate of 2° min^-1^ in the 2*θ* range from 5° to 90°, with Cu Kα radiation (λ=0.15418 nm) at 40 kV and 40 mA. The XRD pole figures were measured using Panalytical X’pert mrd. The morphological images of the samples were recorded by SEM (TESCAN MIRA LMS, Czechia), and energy-dispersive spectrometry (EDS) spectroscopy on a Hitachi S-3500N scanning electron microscope, operated at 200 kV with a resolution of 102 eV. Raman spectra were recorded using LabRAM HR Evolution (HORIBA, France). Absorption spectra were recorded using UV-3600 (SPC, Japan). PL decay dynamics (time-correlated single photon counting) were measured using Edinburgh FLS980 Instrument. A microsecond flash lamp was used to excite samples to monitor the PL decay dynamics in the NIR region. The duration of the pulse for microsecond flash lamp is 7 ns. The inductively coupled plasma optical emission spectrometry (ICP-OES) measurements were conducted using Agilent 725ES emission spectrometer. The photoluminescence quantum yields (PLQYs) were obtained directly by an absolute PL quantum yield measurement system (Hamamatsu Quantaurus-QY). The excitation source is a high-intensity xenon lamp. The excitation wavelength can be changed correspondingly, and the step increments and integration time were 1 nm and 0.5 s per data point, respectively. An integrating sphere was mounted on the spectrofluorometer with the entrance and exit ports set in 90° geometry. The sample was located in the center of the integrating sphere. All the recorded spectroscopic data were corrected for the spectral responses of both the integrating sphere and the spectrofluorometer. The responses of the detecting systems in photon flux were determined using a calibrated tungsten lamp. The experimental wavelength resolution is ±0.1 nm. The temperature-dependent and room temperature down-shifting photoluminescence excitation and emission spectra were recorded by a spectrofluorometer with a temperature controller (Edinburgh FLSP-920). Contact angle was measured using JY-82C. Transmission electron microscopy (TEM) studies were carried out by Talos F200x (FEI, America). The accurate refractive index was measured by QT-TO1000 terahertz imaging system, as displayed in Figure S1.


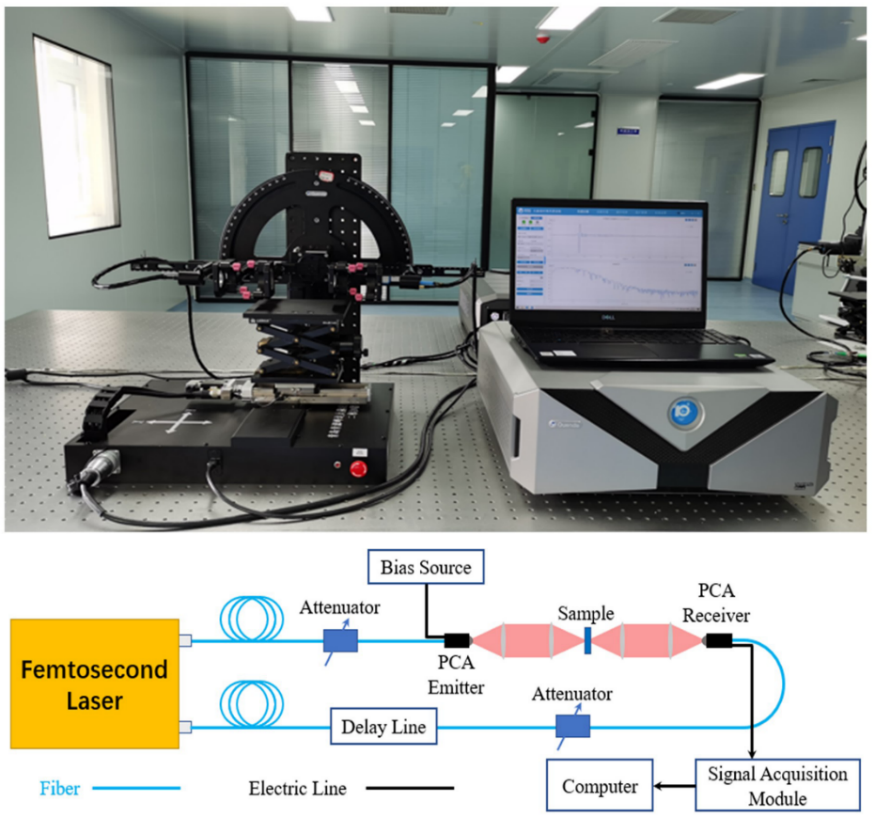


**Figure S1**. Schematic diagram and photo of the experimental setup of QT-TO1000.

***The calculation of the average lifetime, radiative and non-radiative decay rates***: We calculated the radiative recombination rates and non-radiative recombination rates of Cs_2_Ag_x_Na_1-x_ErCl_6_ double perovskites by photoluminescence quantum yields (PLQYs) and the mean lifetimes (*τ_ave_*). The PLQY is the ratio of the number of photons emitted to the number absorbed. Both the radiative recombination and the non-radiative recombination depopulated the excited state. Hence, the PLQY can be also defined as the ratio of the radiative recombination rate to the total recombination rate, given by

*PLQY* **=** $\frac{k_{r}}{k_{r}+k_{nr}}$

in which *k_r_* and *k_nr_* are the radiative recombination rate and the non-radiative recombination rate, respectively. The average lifetime is the inverse of the total recombination rate, given by

$\text{τ}_{ave}$ $\text{=}$ $\frac{1}{k_{r}+k_{nr}}$

where *τ_ave_* is the average time calculated by the fitted data of the time-resolved PL decay. As observed in Ag^+^- or Yb^3+^-doped Cs_2_NaErCl_6_ double perovskites, all the decay lifetimes (at 1540 nm) of the sample show single-exponential function that can be fitted well: I(t) = A𝑒 ^𝑡⁄𝜏^, in which A is the amplitude of component, *τ* is the corresponding lifetime constant (the average lifetime *τ_ave_* is the *τ*). Therefore, we can calculate the radiative and non-radiative recombination rates in the following equations:

$$k_{r}\text{ =} \frac{\text{PLQY}}{\text{τ}_{ave}}$$

$k_{nr}\text{ =} \frac{1}{\text{τ}_{ave}}-k_{r}$ $\text{=}$ $\frac{1-PLQY}{\text{τ}_{ave}}$

***Judd-Ofelt analysis***: The Judd-Ofelt analysis is based on assuming the states are completely degenerate in angular momentum, and the energy denominators are equal. The calculated oscillator strength can be written as

$f_{cal}$(𝑎𝐽, 𝑏𝐽′) = $\frac{\text{8π}\text{2}\text{mc}}{\text{3hλ(2J + 1)e}\text{2}\text{n}\text{2}}$ [$\chi_{ED}S_{ED}$(𝑎𝐽, 𝑏𝐽′) + $\chi_{MD}S_{MD}$(𝑎𝐽, 𝑏𝐽′)]

where χ is local field corrections, for electric dipole transition χ_ED_ = n(n^2^+2)^2^/9 and for magnetic dipole transition χ_MD_ = n^3^. S_ED_ and S_MD_ are the line strength of electric dipole transition and magnetic dipole transition (omitted in our case), respectively. S_ED_ can be expressed as

$$S_{ED}\text{(}\text{aJ}\text{, }\text{bJ}\text{'}\text{)}\text{ }\text{= }\text{e}^{\text{2}}\sum_{\lambda\text{=2,4,6}} \text{Ω}_{\text{λ}}\text{|}\text{<}\text{4}f^{N}aJ\text{‖}\text{U}^{\text{λ}}\text{‖}\text{4}f^{N}bJ^{'}\text{ }\text{>}\text{ }\text{|}^{2}\text{ }$$

where *U^λ^* are the irreducible tensor forms of the dipole operator, *n* is the refractive index of the solid, *λ* is the mean wavelength of the transition, *h* is Planck’s constant, *e* is the electron charge, *m* is the mass and *c* is the speed of light.

Theoretically, it is possible to calculate the Judd-Ofelt parameter, but this requires accurate values for the radial integrals and odd-order crystal field component, which are not known to a high enough degree of precision. Instead, the Judd-Ofelt parameters can be treated as a set of phenomenological parameters to be determined from fitting experimental absorption measurements determined in

$f_{m}$ = $\frac{\text{4}\text{ε}\text{m}\text{c}^{2}}{\text{e}^{2}\text{λ}^{2}}$ $\int\text{σ}_{abs}\text{(}\text{λ}\text{)}\text{d}\text{ }\text{λ}\text{ = }\frac{\text{4}\text{ε}\text{m}\text{c}^{2}}{\text{e}^{2}\text{λ}^{2}} \text{(2.303 ×}$ $\int\text{log (}\frac{I_{\text{0}}}{\text{I}}\text{)}$)

Where σ_abs_(λ) is the wavelength dependent absorption cross section. Once the Judd-Ofelt parameters are determined, they can be used to calculate transition probabilities, *A(J; J’)*, of all excited states from the following equation,

𝐴(𝐽′; 𝐽) = $\frac{\text{64}\text{π}^{4}\text{e}^{2}}{\text{3}\text{h}\text{ }\text{(2}\text{J'}\text{ + 1) }{\bar{\text{λ}}}^{3}}$ [$\text{n }{\text{(}\frac{\text{n}^{2}+2}{3}\text{)}}^{2}S_{ED}$]

***Molecular dynamics simulation***: Classic molecular dynamics (MD) simulations were carried out to investigate the confined systems from the atomic level for two cases (system 220, system 222). System 220 contains 5560 H_2_O and one (220) facet. System 222 contains 5560 H_2_O and one (222) facet. The initial configuration systems were constructed through the software of PACKMOL.^(2)^ The Tip3p force field was employed to describe the H_2_O.^(3)^ The UFF force field was employed to describe the facets.^(4)^ For different kinds of atoms, the Lorentz-Berthelot mix rules were adopted for van der Waals (vdW) interactions. The cutoff distance of vdW and electronic interactions was set to 1.2 nm. For the simulation, an energy minimization was firstly employed to relax the simulation box. Then, a canonical (NVT) ensemble with a 1.0 fs time step is employed to optimize the simulation water droplet, where the temperature is set to 300 K. The temperature was kept via the Nose-Hoover thermostat. The NVT optimization time was set to 10.0 ns. In all the MD simulations, the motion of atoms was described by classical Newton’s equation, which was solved using the Velocity-Verlet algorithm. And all the MD simulations were performed by using LAMMPS 2022.6 package.^(5)^

**Experimental Results**

**Table S1.** Crystallographic parameters obtained from XRD Rietveld refinement.


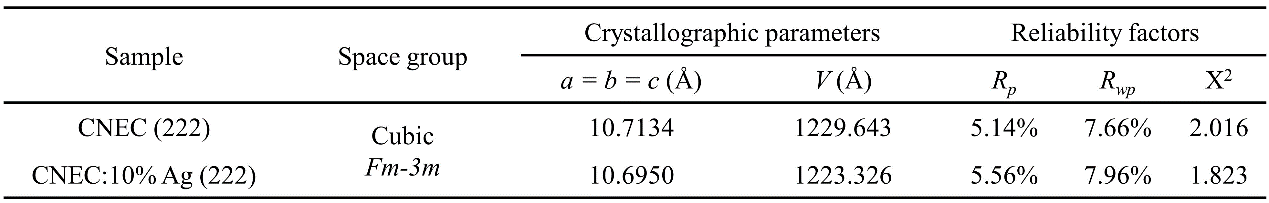


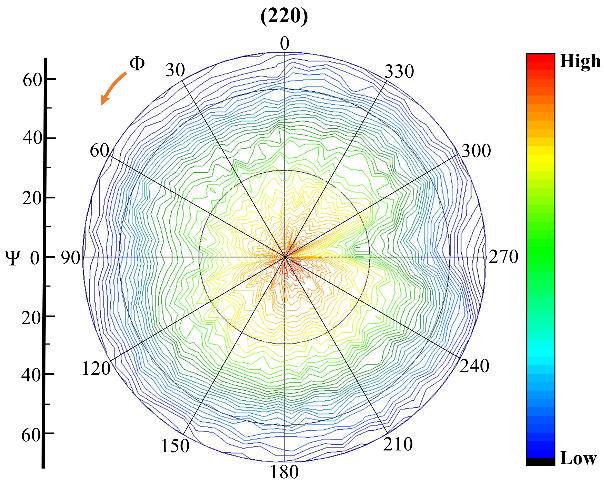


**Figure S2**. Normalized (220) pole figure of pristine CNEC (222).


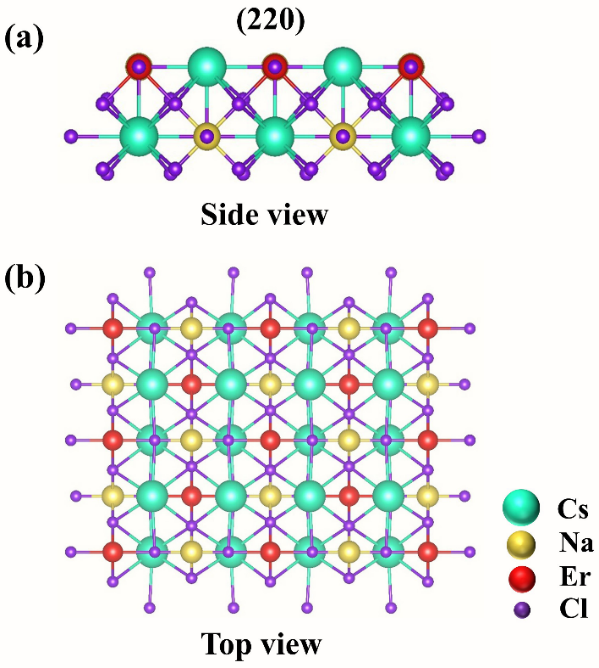


**Figure S3**. Crystal structure of CNEC (220) in side view and top view.

**Table S2.** Inductively coupled plasma optical emission spectrometer (ICP-OES) data of Ag-alloyed CNEC (222) with different doping concentrations.


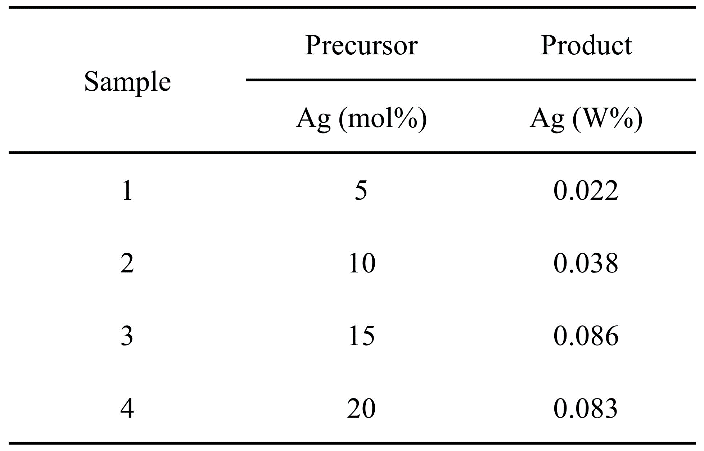


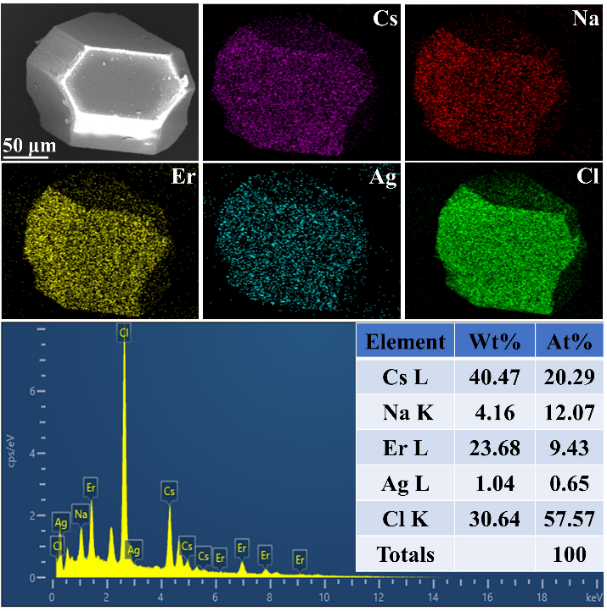


**Figure S4**. SEM image and EDS distribution of the elements in CNEC:10% Ag (222).


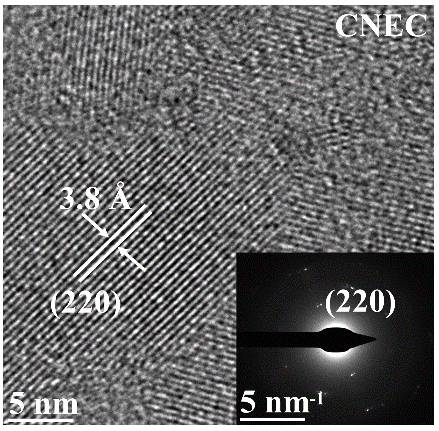


**Figure S5**. HRTEM image and SAED pattern of CNEC (220).


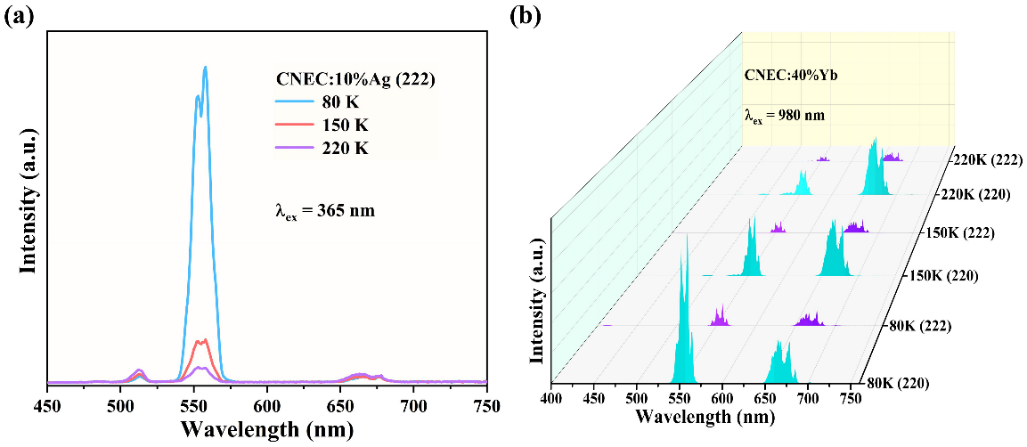


**Figure S6**. Low-temperature PL spectra of (a) CNEC:10% Ag (222) under 365 nm excitation and (b) CNEC:40% Yb (220) and CNEC:40% Yb (222) under 980 nm excitation.


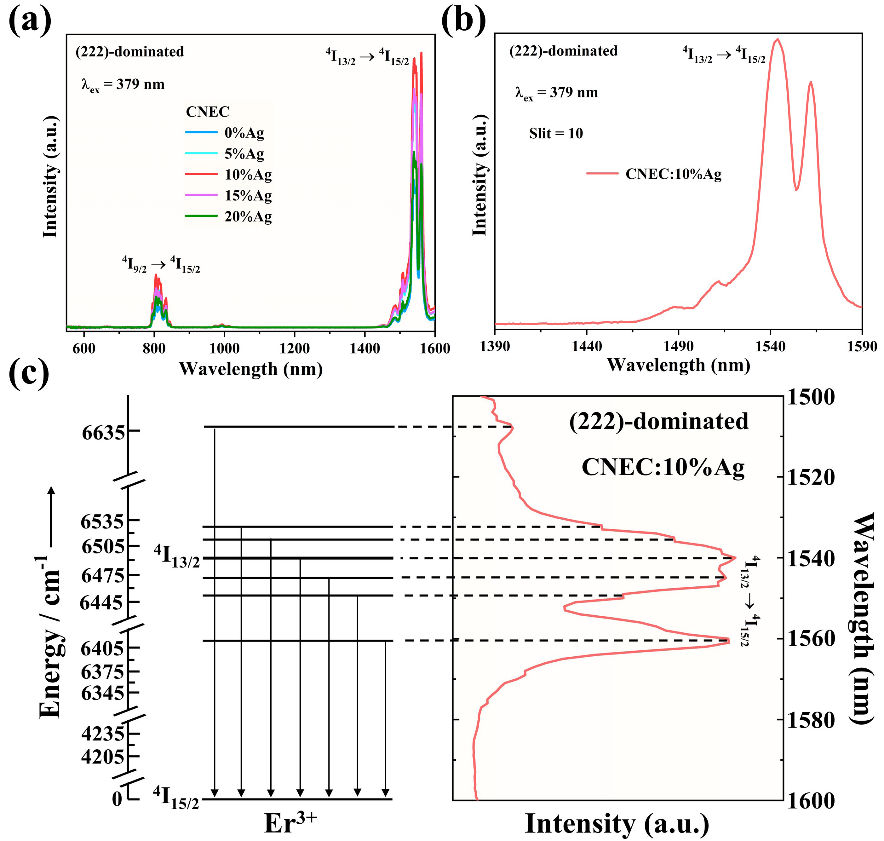


**Figure S7**. (a) PL spectra of (222)-dominated CNEC samples with different Ag^+^ contents under 379 nm excitation. (b) Low-resolution PL spectrum of CNEC:10% Ag (222) (c) Stark splitting ^4^I_13/2_ sublevels of CNEC:10% Ag (222) from high-resolution PL spectra.

**Table S3.** The fitting results of PL decay time for Cs_2_NaErCl_6_ (222) with undoped and Ag^+^-doped samples at 1540 nm under 365 nm excitation.


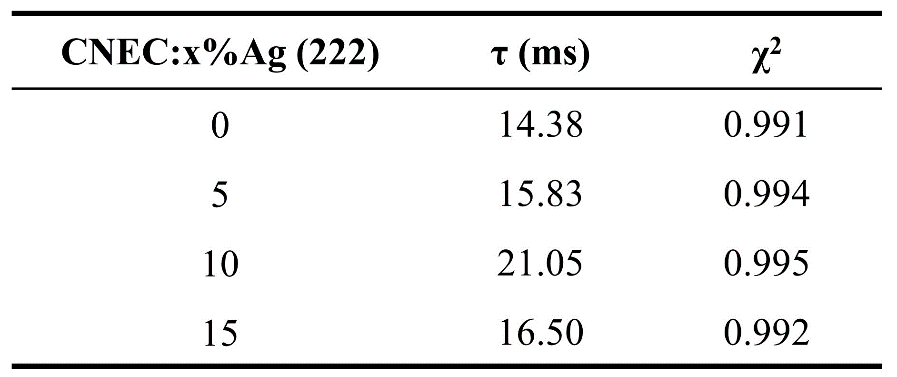


**
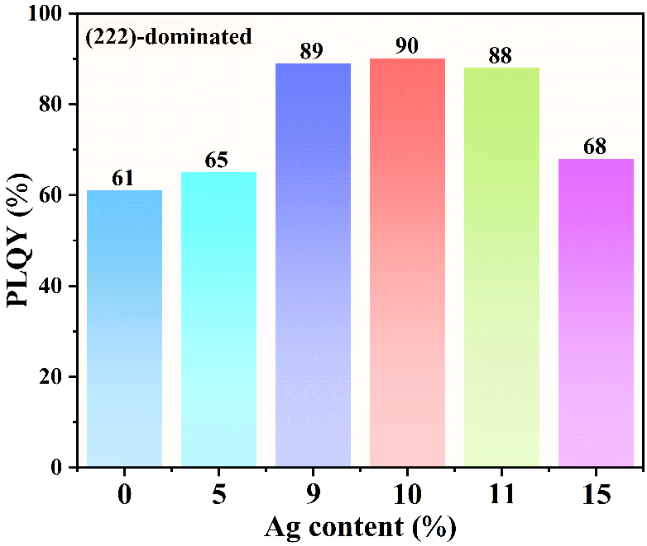
**

**Figure S8**. PLQY results of (222)-dominated CNEC samples with different Ag^+^ contents under 379 nm excitation.

**
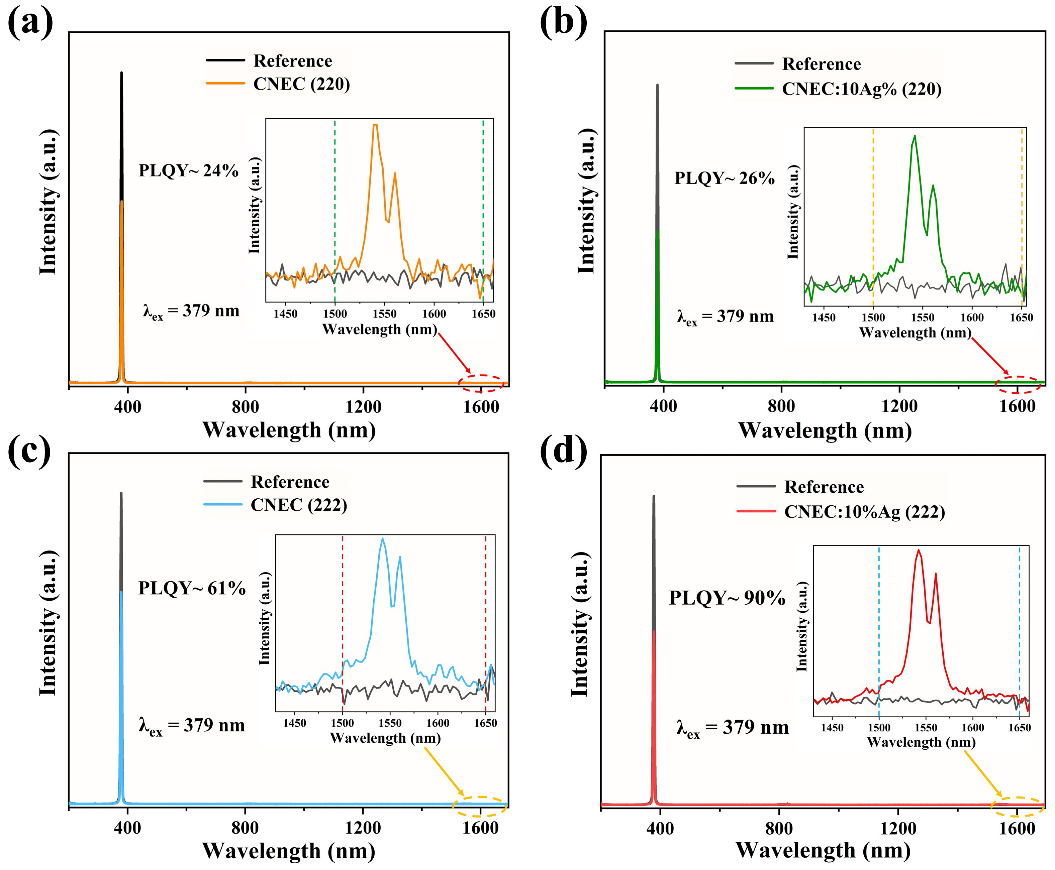
**

**Figure S9**. PLQY results of pristine CNEC (220), CNEC:10% Ag (220), CNEC (222) and CNEC:10% Ag (222) under 379 nm excitation.


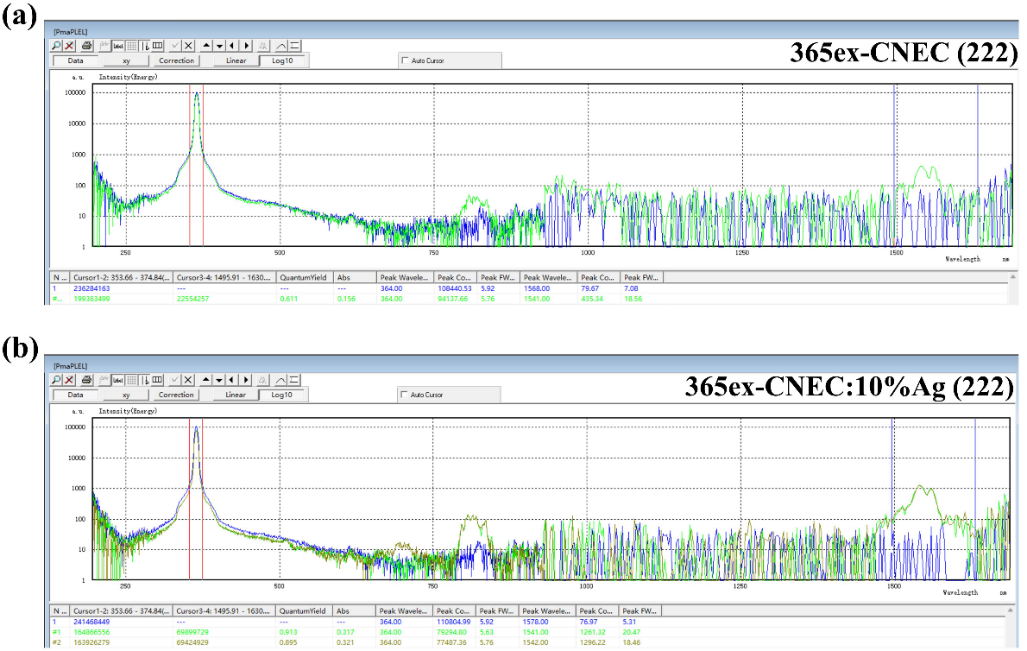


**Figure S10**. Original PLQY results of pristine CNEC (222) and CNEC:10% Ag (222) under 365 nm excitation.


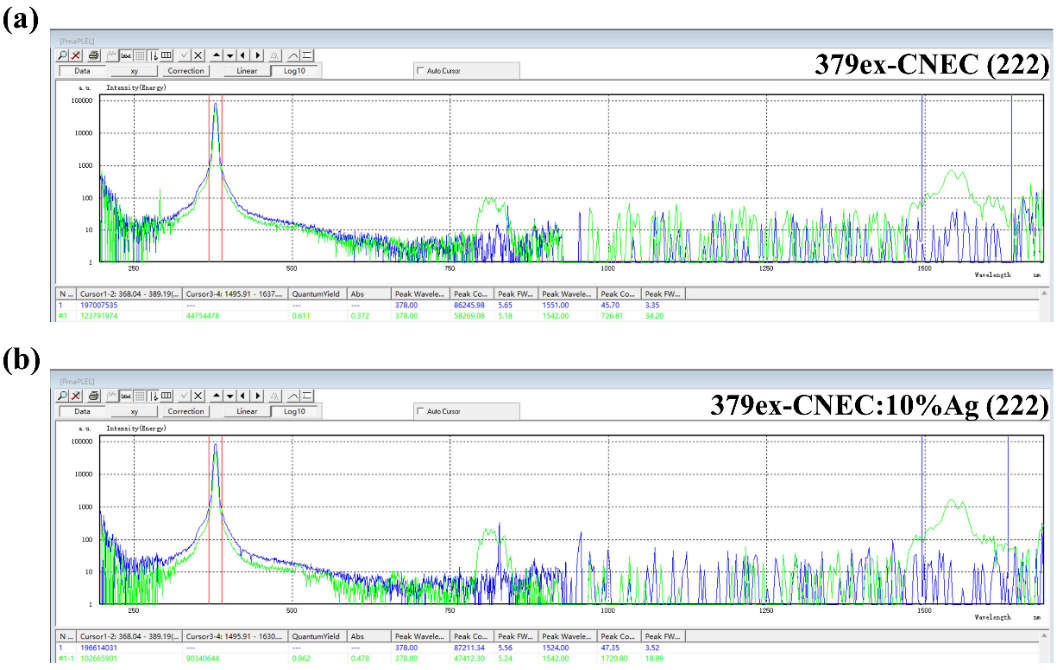


**Figure S11**. Original PLQY results of pristine CNEC (222) and CNEC:10% Ag (222) under 379 nm excitation.


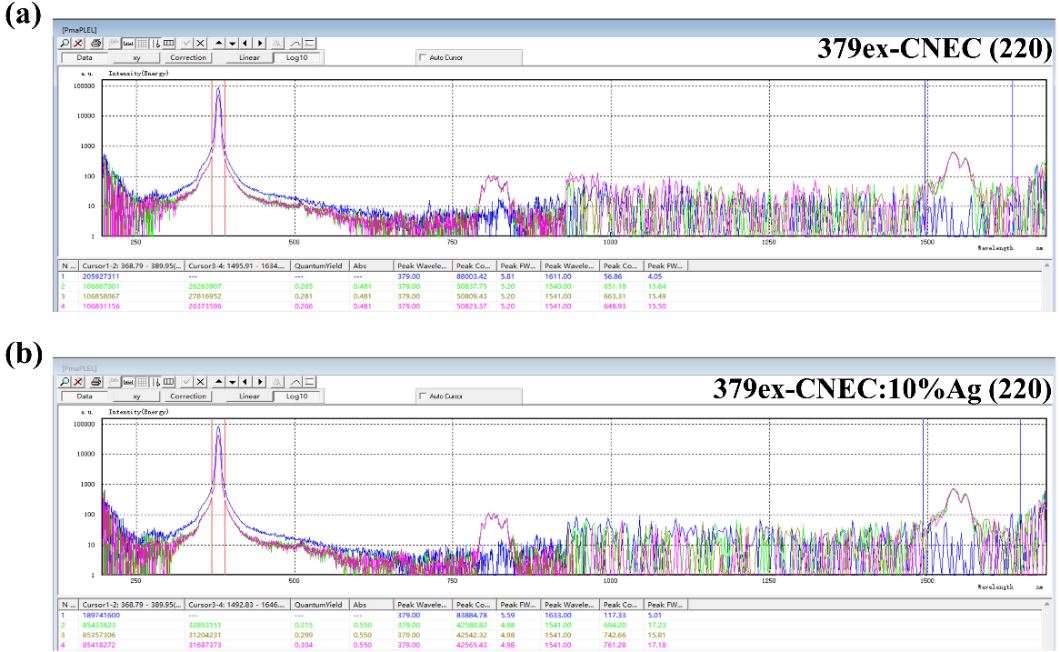


**Figure S12**. Original PLQY results of pristine CNEC (220) and CNEC:10% Ag (220) under 379 nm excitation.

**
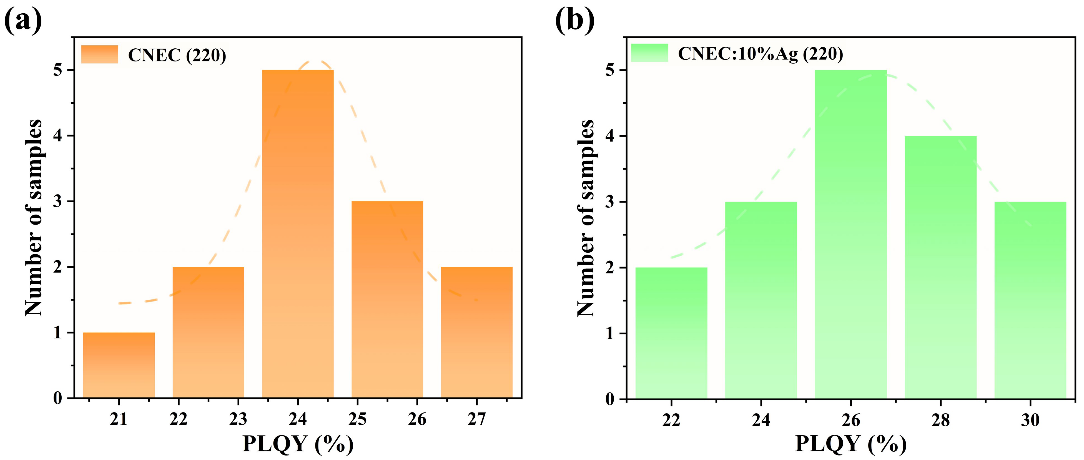
**

**Figure S13**. The statistical PLQY values for pristine CNEC (220) and CNEC:10% Ag (220) under 379 nm excitation, respectively.

**
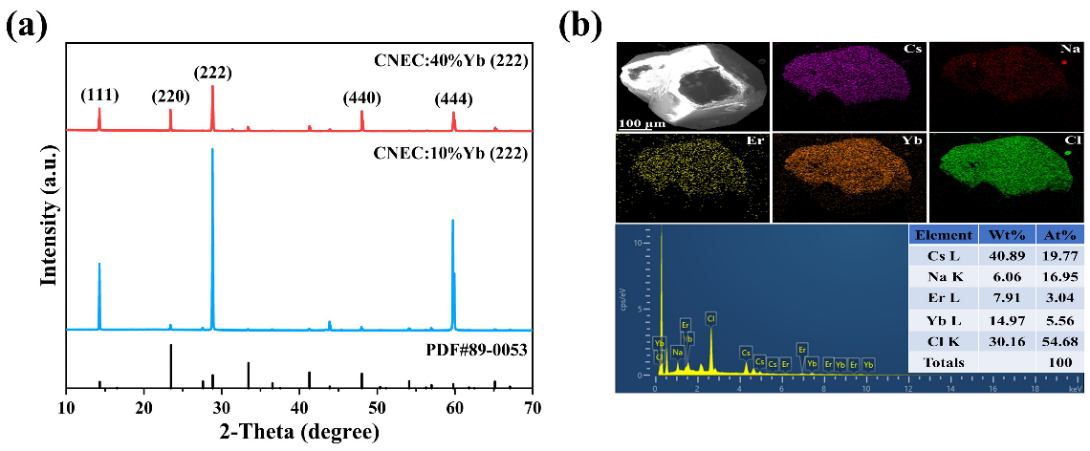
**

**Figure S14**. XRD patterns of 10% and 40% Yb^3+^-doped CNEC (222). (b) SEM image and EDS distribution of the elements in CNEC:60% Yb (222).


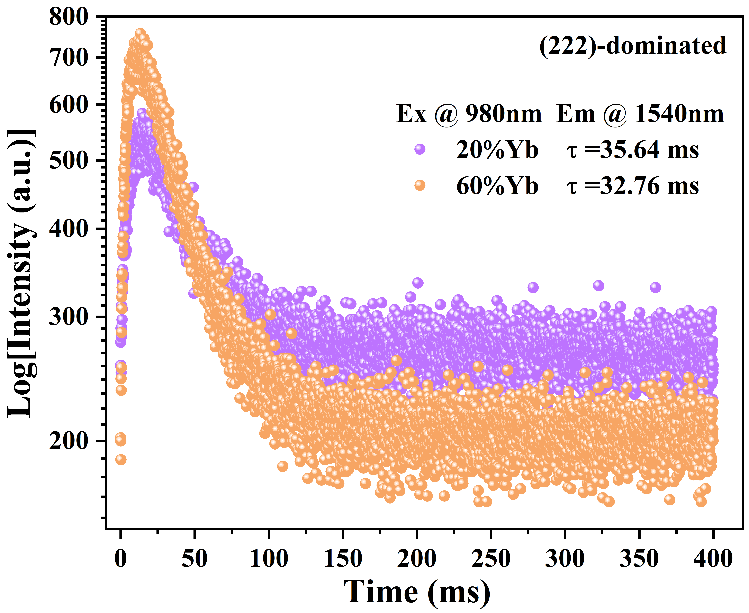


**Figure S15**.The PL decay curves of CNEC:20% Yb (222) and CNEC:60% Yb (222) under 980 nm excitation.

**Table S4.** The fitting results of PL decay time for Cs_2_NaErCl_6_ (222) with undoped and Yb^3+^-doped samples at 1540 nm under 980 nm excitation.

**
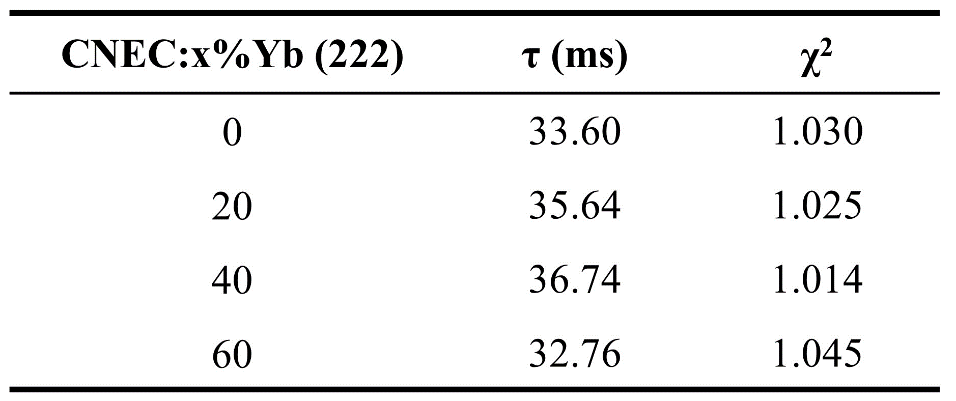
**

**
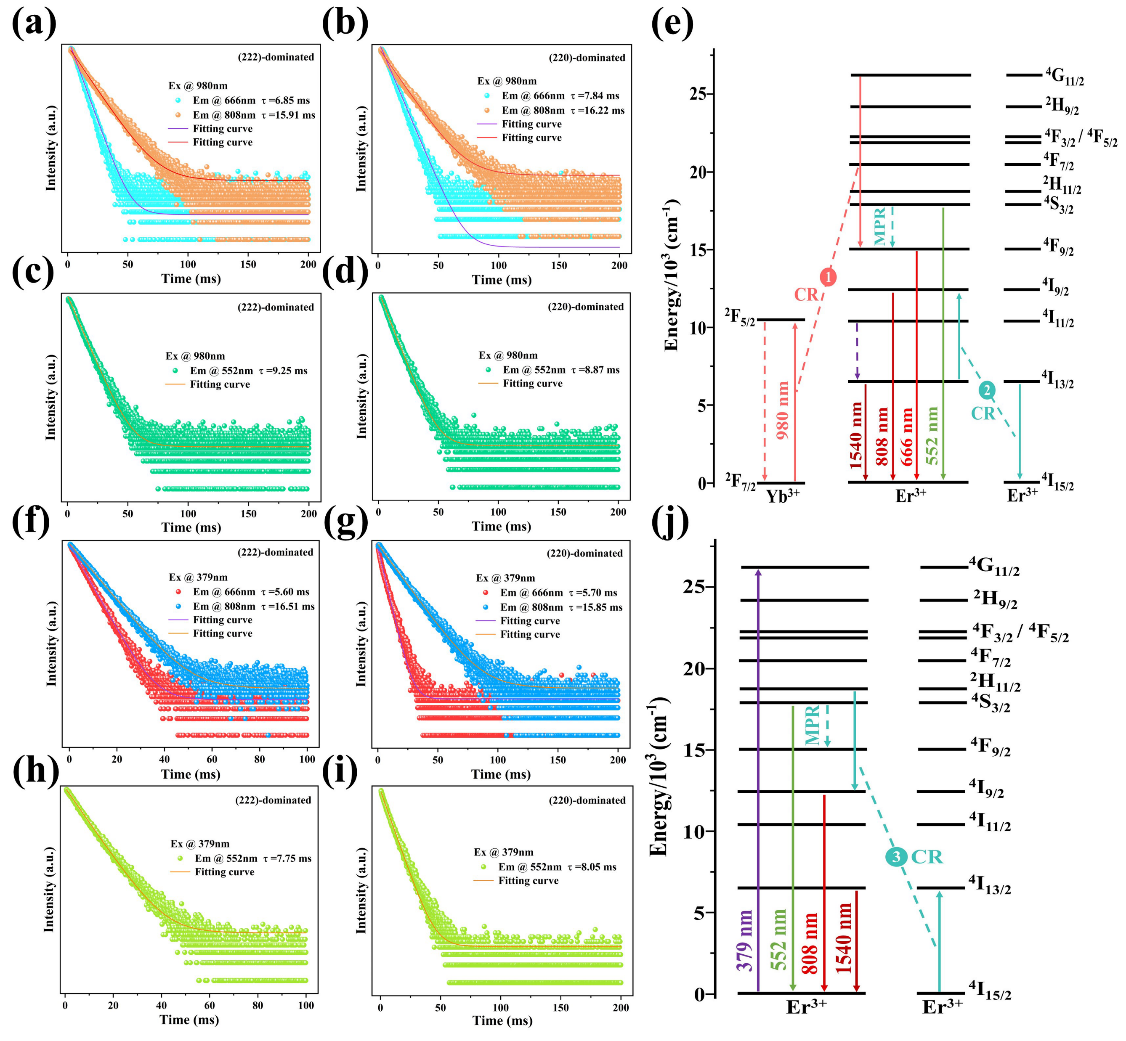
**

**Figure S16**. The PL decay curves of ^4^S_3/2_ (552 nm), ^4^F_9/2_ (666 nm) and ^4^I_9/2_ (808 nm) under 980 nm excitation for (a) and (c) CNEC:40% Yb (222). (b) and (d) CNEC:40% Yb (220). (e) Photophysical processes for three emissions and the proposed mechanism under 980 nm excitation. The PL decay curves of ^4^S_3/2_ (552 nm), ^4^F_9/2_ (666 nm) and ^4^I_9/2_ (808 nm) under 379 nm excitation for (f) and (h) CNEC:40% Yb (222). (g) and (i) CNEC:40% Yb (220). (j) Photophysical processes for three emissions and the proposed mechanism under 379 nm excitation.

**Note**: The PL decay curves at different emission wavelength were measured to better understand the excited-state dynamics in Cs_2_NaErCl_6_ DPs. The PL decays of ^4^S_3/2_ (552 nm, green emission), ^4^F_9/2_ (666 nm, red emission) and ^4^I_9/2_ (808 nm, NIR-Ⅰ emission) to ^4^I_15/2_ transitions of Er^3+^ under 379 and 980 nm excitation have been shown in Figure S16. In the upconversion system (980 nm excitation), for green emissions, the fitted lifetimes of both CNEC:40% Yb (222) and CNEC:40% Yb (220) show single-exponential function (Table S5), and the longer lifetime of (222) facets demonstrate that the nonradiative processes are suppressed by facet strategy. Because the measured lifetime (τ_ave_) in PL decay curve is connected to the radiative (τ_r_) and nonradiative lifetimes (τ_nr_) by the relation 1/τ_ave_ = 1/τ_r_ + 1/τ_nr_. If the main contribution of emission enhancement comes from the suppression of nonradiative processes, it should lead to a prolonged emission lifetime (τ_nr_) for the longer τ_ave_. For red emissions, the lifetime of CNEC:40% Yb (222) (6.85 ms) is shorter than that of (220) counterpart (7.84 ms), indicating the obvious suppression of cross-relaxation (CR-1) process on (222) facet (Figure S16c).^(15)^ For 808 nm emissions, the lifetimes for both are well fitted to bi-exponential function. The fast lifetime component is related to nonradiative processes and the slow lifetime component is associated to 808 nm emission. As shown in Table S5, the longer lifetime (τ_1_=5.84 ms) of fast component on (222) facet proves the enhancement of CR-2 process compared with (220) facet due to the increased electron populations of ^4^I_9/2_ (Figure S16c); while the shorter lifetime (τ_2_=17.43 ms) of slow component on (222) facet means that lower local symmetry would facilitate electronic transitions, and increases the radiative transition rate (k_r_=1/τ_2_), which is closely related to local symmetry. Totally, τ_ave_ of (222) facet (15.91ms) is shorter than that of (220) facet (16.22 ms), indicating that the radiative transition rate k_r_ plays a predominant role for brighter 808 nm luminescence, as a result of the breaking of local symmetry.^(16)^ The results demonstrate that 808 nm emission is more sensitive to local symmetry compared with green and red emissions.

In the downshifting system (379 nm excitation), for green emissions, the fitted lifetimes of both CNEC:40% Yb (222) and CNEC:40% Yb (220) show bi-exponential function (Table S5). The fast lifetime component (τ_1_) for two facets is in the microsecond scale, indicating the dominated multi-phonon relaxation (MPR) process.^(17)^ The shorter lifetime (τ_2_=7.89 ms) of slow component on (222) facet also means that its lower local symmetry would facilitate green emission compared to (220) counterpart. For red emissions, the average lifetime of (222) sample is shorter than that of (220) counterpart, indicating the slightly suppression of CR-1 process on (222) facet. For 808 nm emissions, the lifetimes for both are well fitted to mono-exponential function, and the longer lifetime of (222) facets demonstrate that the CR-3 process is dominated related to 808 nm emission (Figure S16f).

**Table S5.** The fitting results of PL decay time for CNEC:40% Yb (220) and CNEC:40% Yb (222) at 552 nm, 666 nm and 808 nm under 980 nm or 379 nm excitation, respectively.


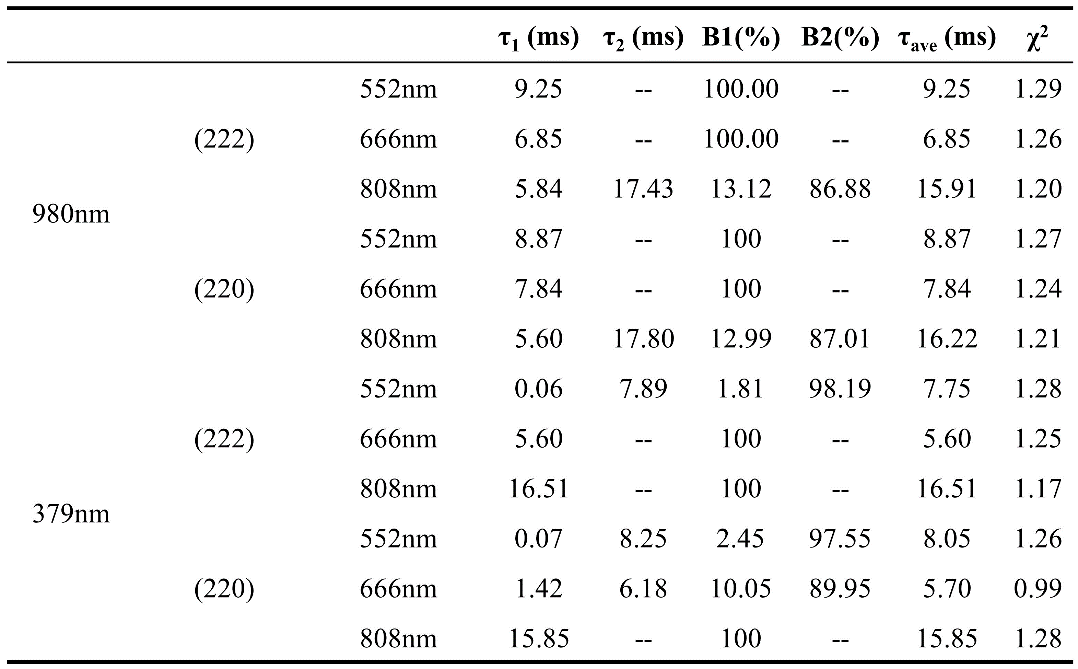


**
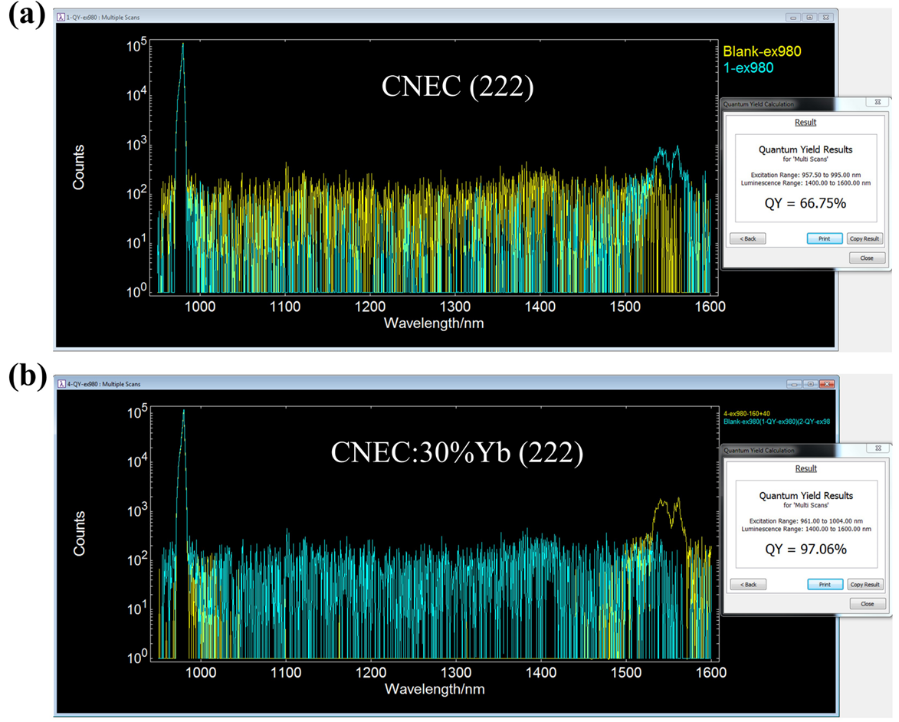
**

**Figure S17**. Original PLQY results of pristine CNEC (222) and CNEC:30% Yb (222) under 980 nm excitation.

**
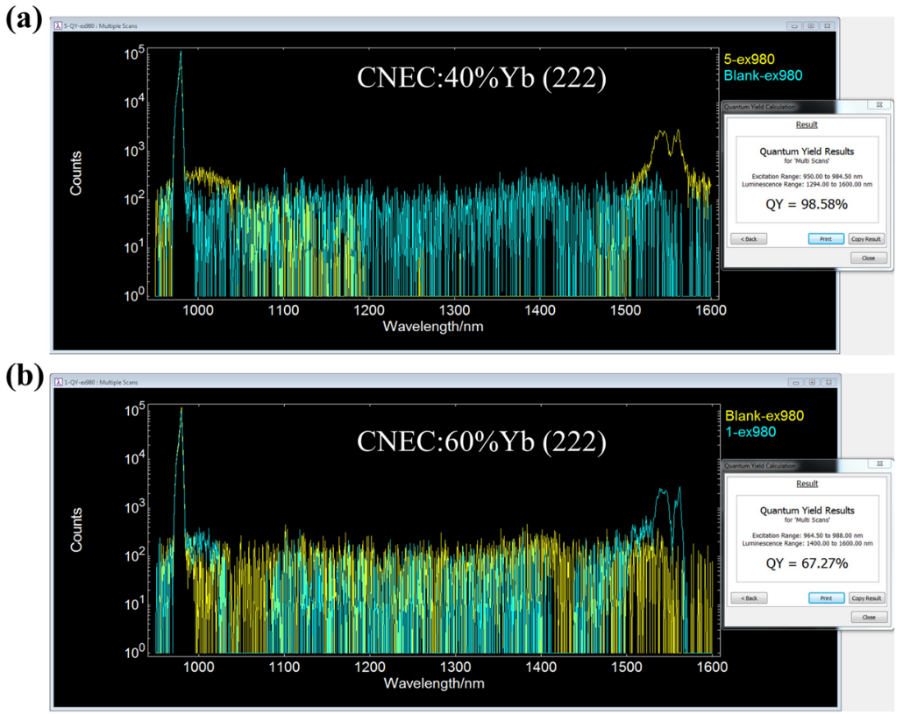
**

**Figure S18**. Original PLQY results of CNEC:40% Yb (222) and CNEC:60% Yb (222) under 980 nm excitation.

**Table S6.** Some PLQYs for 1540 nm emissions based on reported halide double perovskites.

**
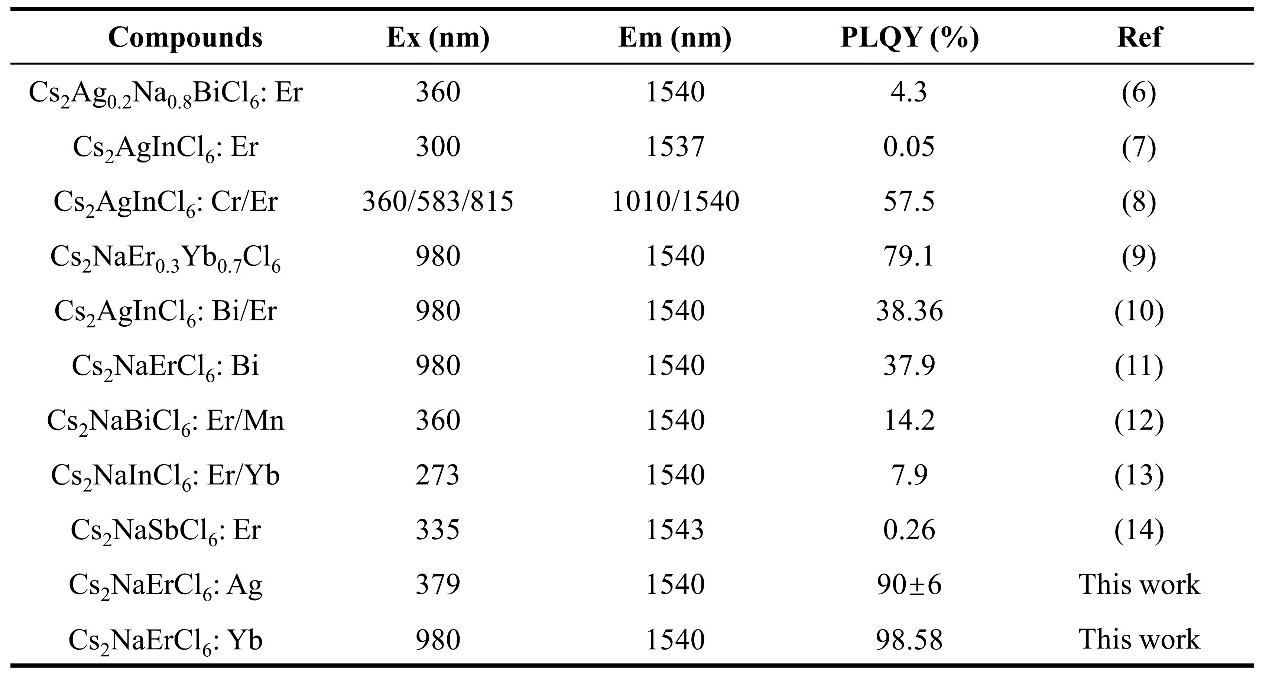
**


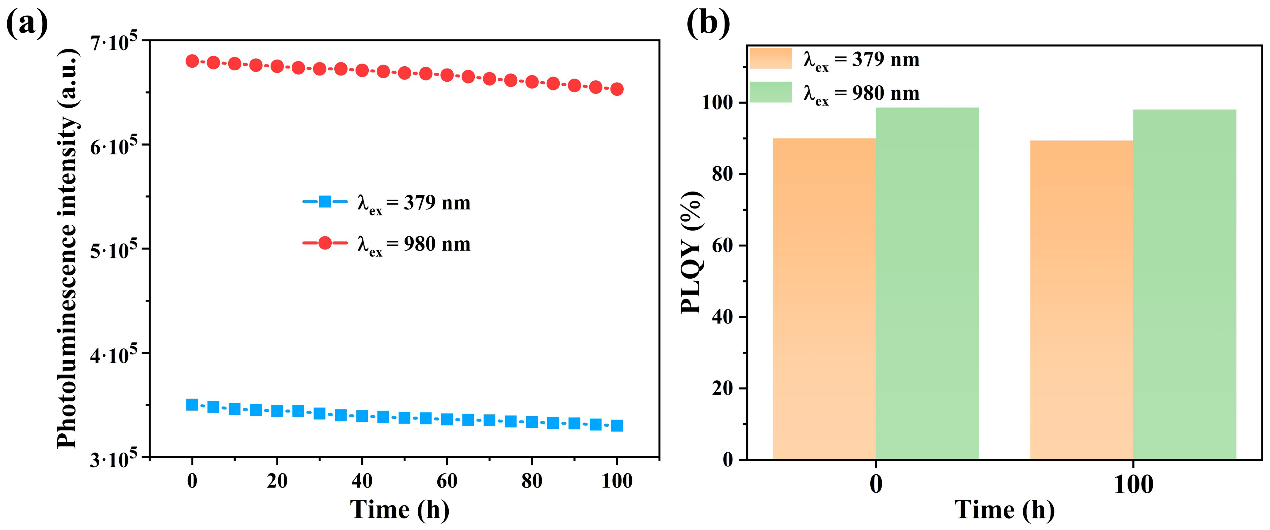


**Figure S19**. (a) The operational stability of CNEC:20% Yb (222) under continuous illumination with 379 nm and 980 nm over 100 hours. (b) The corresponding PLQY values before and after continuous illumination.

**Table S7.** J-O results for pristine CNEC (220), CNEC (222) and CNEC:10% Ag (222).


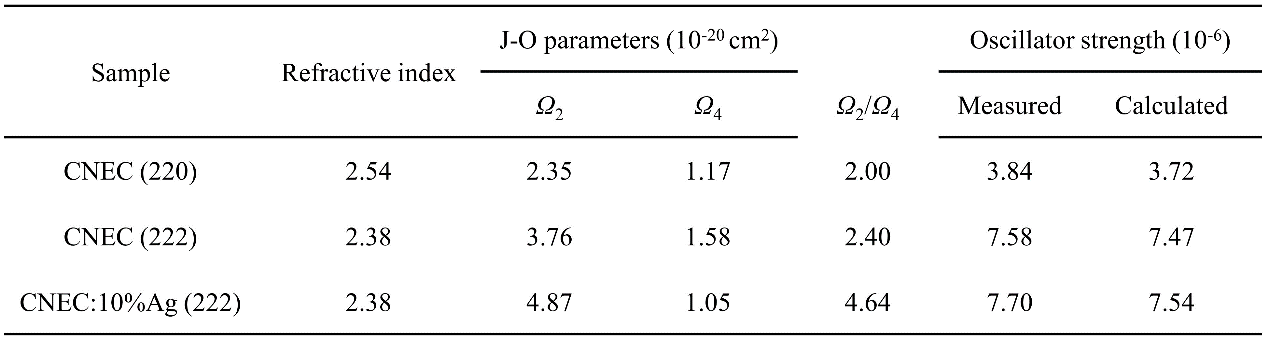


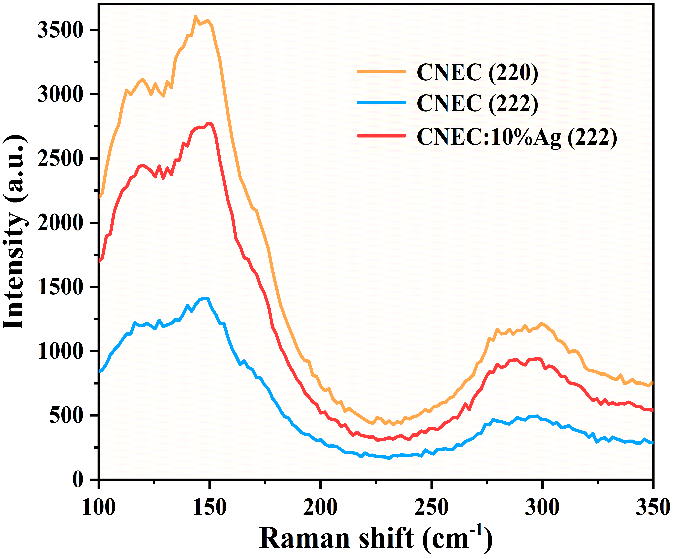


**Figure S20**. Raman spectra of pristine CNEC (220), CNEC (222) and CNEC:10% Ag (222).


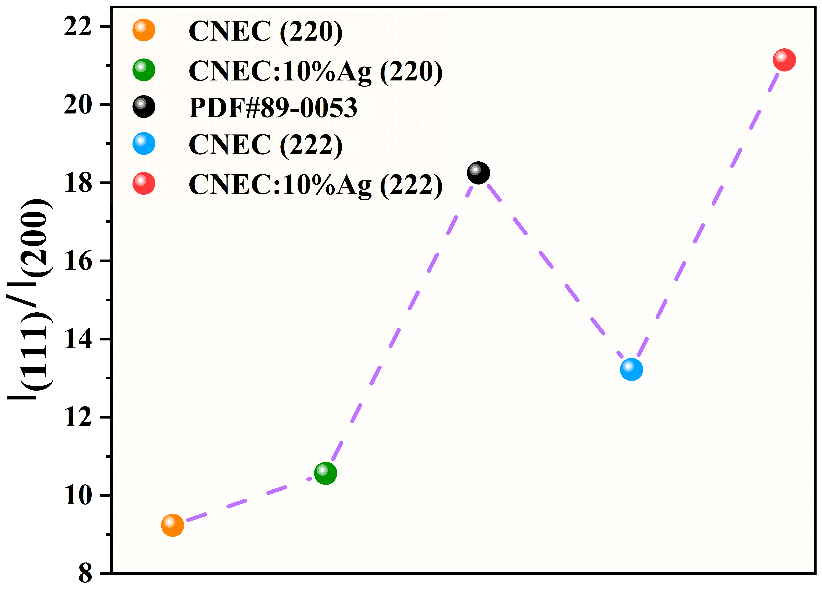


**Figure S21**. The relative intensity ratio of (111) and (200) peak (I_111_/I_200_) of pristine CNEC (220), CNEC:10% Ag (220), standard pattern of Cs_2_NaErCl_6_, CNEC (222) and CNEC:10% Ag (222).

**
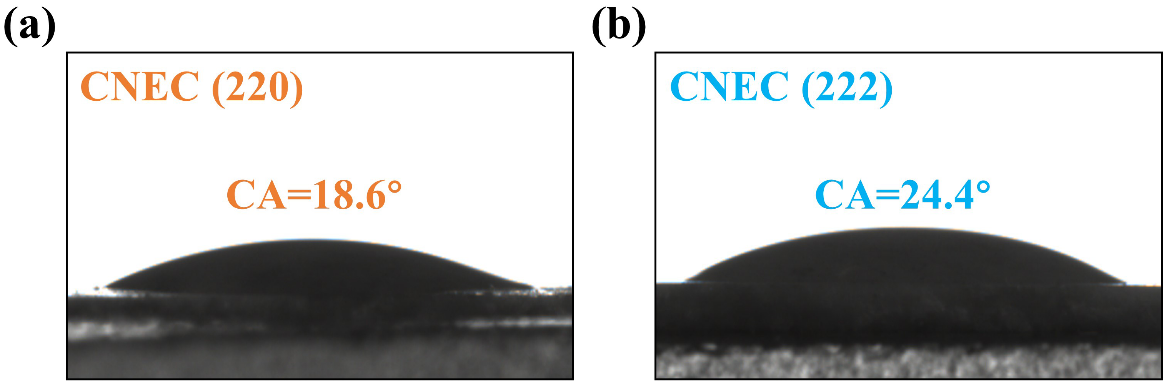
**

**Figure S22**. The contact angle measurement of (a) CNEC (220) and (b) CNEC (222).

**References**

(1) J. Luo, X. Wang, S. Li, J. Liu, Y. Guo, G. Niu, L. Yao, Y. Fu, L. Gao, Q. Dong, C. Zhao, M. Leng, F. Ma, W. Liang, L. Wang, S. Jin, J. Han, L. Zhang, J. Etheridge, J. Wang, Y. Yan, E. H. Sargent, J. Tang, *Nature* **2018**, *563*, 541.

(2) L. Martínez, R. Andrade, E. G. Birgin, J. M. Martínez, *J. Comput. Chem.* **2009**, *30*, 2157.

(3) https://doi.org/10.25950/962b4967; https://doi.org/10.25950/ac258694; 10.1007/s11837-011-0102-6; 10.25950/ff8f563a.

(4) W. L. Jorgensen, J. Chandrasekhar, J. D. Madura, R. W. Impey, M. L. Klein, *J. Chem. Phys.* **1983**, *79*, 926.

(5) S. Plimpton, *J. Comput. Phys.* **1995**, *117*, 1.

(6) Y. Pei, D. Tu, C. Li, S. Han, Z. Xie, F. Wen, L. Wang, X. Chen, *Angew. Chem. Int. Ed.* **2022**, *61*, e202205276.

(7) W. Lee, S. Hong, S. Kim, *J. Phys. Chem. C* **2019**, *123*, 2665.

(8) W. Gan, L. Cao, S. Gu, H. Lian, Z. Xia, J. Wang, *Chem. Mater.* **2023**, *35*, 5291.

(9) Y. Zhu, G. Pan, J. Zhao, K. Liu, W. Xue, Y. Wang, W. You, H. Gao, W. Xu, Y. Mao, *Adv. Optical Mater.* **2022**, *11*, 2202019.

(10) J. Zhao, G. Pan, K. Liu, W. You, S. Jin, Y. Zhu, H. Gao, H. Zhang, Y. Mao, *J. Alloys Compd.* **2022**, *895*, 162601.

(11) X. Xu, P. Han, D. Zheng, K. Du, C. Li, F. Liu, R. Zhang, K. Han, *Laser Photonics Rev.* **2022**, *16*, 2200318.

(12) W. Gan, B. M. Liu, L. Huang, S. Lou, J. Zhang, Z. Zhou, J. Wang, *Adv. Opt. Mater.* **2022**, *10*, 2102851.

(13) S. Han, D. Tu, Z. Xie, Y. Zhang, J. Li, Y. Pei, J. Xu, Z. Gong, X. Chen, *Adv. Sci.* **2022**, *9*, 2203735.

(14) R. Wu, P. Han, D. Zheng, J. Zhang, S. Yang, Y. Zhao, X. Miao, K. Han, *Laser Photonics Rev.* **2021**, *15*, 2100218.

(15) G. Zhang, P. Dang, H. Lian, H. Xiao, Z. Cheng, J. Lin, *Laser Photonics Rev.* **2022**, *16*, 2200078.

(16) Q. Duan, Y. Xu, R. Yang, D. Hong, D. Zhou, Q. Wang, Y. Yang, J. Han, Y. Wen, J. Qiu, *Inorg. Chem. Front.* **2024**, *11*, 246.

(17) R. Shi, A.-V. Mudring, *ACS Materials Lett.* **2022**, *4*, 1882.
